# Uterine Stroma-Derived Tumors and the Extracellular Matrix: A Comparative Review of Benign and Malignant Pathologies

**DOI:** 10.3390/cancers17213501

**Published:** 2025-10-30

**Authors:** Maria Marmara, Thomas Vrekoussis, Fanourios Makrygiannakis, Dragana Nikitovic, Aikaterini Berdiaki

**Affiliations:** 1 Department of Histology-Embryology, Medical School, University of Crete, 70013 Heraklion, Greece; bio3199@edu.biology.uoc.gr (M.M.); medp2012152@med.uoc.gr (F.M.); nikitovic@uoc.gr (D.N.); 2 Laboratory of Human Reproduction, Department of Obstetrics and Gynecology, Medical School, University of Crete, 70013 Heraklion, Greece; thomas.vrekousis@uoc.gr

**Keywords:** extracellular matrix, uterine stromal tumors, uterine sarcoma, signaling pathways, cancer therapy, tumor microenvironment

## Abstract

**Simple Summary:**

Uterine stromal-derived tumors, rare neoplasms, range from benign endometrial stromal nodules to aggressive high-grade endometrial stromal sarcomas and undifferentiated uterine sarcomas. Endometrial stromal tumors localize in the hormone-responsive connective tissue of the endometrium, the uterus’s inner layer. This review summarizes the classification of these tumors based on molecular and immunohistochemical profiling. Furthermore, the existing knowledge on the role of the extracellular matrix in the biology of endometrial stromal tumors is described. Although ECM’s importance in the normal uterus has been widely studied, its role in endometrial cancer is limited. Modulation of ECM molecules, including collagens, proteoglycans, and glycosaminoglycans, as well as matricellular enzymes, has been shown to remodel stromal architecture and influence tumor formation, function, and cancer development. The presented evidence suggests that ECM molecules may serve as diagnostic and prognostic biomarkers, as well as targets for therapy in uterine sarcomas. Understanding the ECM-driven mechanisms of these challenging tumors may ultimately improve their classification, prognosis and treatment.

**Abstract:**

Uterine stromal-derived tumors encompass a spectrum of rare neoplasms, ranging from benign endometrial stromal nodules to aggressive high-grade endometrial stromal sarcomas and undifferentiated uterine sarcomas. The classification of these tumors has advanced through molecular and immunohistochemical profiling, but the role of the extracellular matrix (ECM) in their biology is only beginning to be understood. The ECM provides both structural support and dynamic signaling cues, regulating tumor cell proliferation, invasion, angiogenesis, and immune evasion. Altered expression of collagens, proteoglycans, glycosaminoglycans, and matricellular proteins reshapes stromal architecture and contributes to disease progression. Moreover, ECM remodeling enzymes such as matrix metalloproteinases, together with cross-linking factors, create a stiff and pro-tumorigenic microenvironment that facilitates invasion and therapeutic resistance. Furthermore, these matrix alterations intersect with angiogenesis, mechanotransduction pathways, and immune modulation. Studies to date describe the role of ECM molecules in the function of the physiological uterine tissue and data for the uterine stroma-derived tumors is scarce. This review summarizes the existing knowledge in classification, prognosis and diagnosis, and summarizes the ECM-driven mechanisms in tumors described so far, aiming to identify new and prognostic biomarkers and novel therapeutic targets in uterine sarcomas.

## 1. Introduction

Endometrial cancer is the most common type of female reproductive cancer and an important source of morbidity and mortality [1]. Endometrial stromal tumors (ESTs) are a rare, mesenchymal subtype of endometrial cancer, emerging from the hormone-responsive connective tissue of the endometrium. ESTs are presented in the rate of 0.30 per 100,000 women [2], but despite their low annual incidence, they are accompanied by serious diagnostic and management challenges. Notable for their diverse morphology, behavior and genetic composition, they are divided into four different subtypes: Endometrial Stromal Nodules (ESNs), Low-Grade Endometrial Stromal Sarcomas (LG-ESSs), High-Grade Endometrial Stromal Sarcomas (HG-ESSs) and Undifferentiated Uterine Sarcomas (UUSs) [3]. Mortality rates vary and depend on the type of EST and the stage at diagnosis. ESNs, as benign tumors, have a near-zero mortality rate [4]. LG–ESS also demonstrates a favorable outcome, with a mortality rate less than 10% in early-stage diagnosis [5]. On the other hand, HG-ESS patients have significantly lower survival chances, with a mortality rate up to 67.2% [6]. Similarly, UUSs are associated with a concerning mortality outcome of 75% [7]. Accurate diagnosis is essential because of differences in clinical outcomes and management strategies. Lower-grade ESTs display indolent clinical progression and are commonly hormone-responsive, enabling the use of simpler treatment options, like surgical removal and hormonal therapy. In contrast, higher-grade stromal tumors present an aggressive clinical course accompanied by low expression or absence of estrogen and progesterone receptors [4]. These facts, along with the common development of resistance to conventional therapies, underscore the need for modern, more efficient treatments. The role of endometrial tumor microenvironment (TME) and its extracellular matrix (ECM) in the signaling and development of endometrial cancer is just beginning to be valued and it is rendered crucial to be the subject of future EST involved studies. In light of these points, we aim to provide a comprehensive overview of current knowledge on different types of ESTs and to compare their features, integrating pathological findings with molecular and ECM-related perspectives. This review will serve as a resource for future investigations, enabling researchers to build on established findings to explore novel molecular pathways and therapeutic targets.

## 2. Classification and Histopathology

Endometrial stromal tumors (ESTs) form a complex subset of uterine neoplasms with heterogeneous morphological and immunohistochemical characteristics. Based on their different features, ESTs are sorted in four main categories: Endometrial Stromal Nodules (ESNs), Low-Grade Endometrial Stromal Sarcomas (LG-ESSs), High-Grade Endometrial Stromal Sarcomas (HG-ESSs), and Undifferentiated Uterine Sarcomas (UUSs) [3].

### 2.1. Classification and Histopathology of Endometrial Stromal Nodules (ESNs)

The term ESNs refers to a group of benign uterine stromal tumors. Macroscopically, it presents as a well-circumscribed mass of variable size, with clear, expansive and non-infiltrative margins. It can be intramural or submucosal, with a soft, yellow cut surface. Hemorrhage, necrosis, and cystic degeneration are occasional findings [8]. Myometrial and lymphovascular invasion are absent. Prominent arterioles are usually formed. ESNs consist of uniform cells that resemble the proliferative phase of the menstrual cycle. Typically, they are densely cellular, with oval nuclei and scanty cytoplasm. Minimal cytological atypia is noticed, and the mitotic rate is low [3,9]. The surrounding stroma is usually non-fibrotic and maintains an ECM composition similar to that of the normal endometrium, which helps distinguish ESNs from infiltrative sarcomas [10].

### 2.2. Classification and Histopathology of Low-Grade Endometrial Stromal Sarcomas (LG-ESSs)

LG-ESSs are defined as slow-growing sarcomas with endometrial stromal origin. This group shows the same histopathological features as ESNs, except for infiltrative and permeative margins. On gross examination, submucosal or intramural neoplasms with ill-defined borders are observed. They present either as confluent soft tan to yellow masses or worm-like tumor plugs [8]. Extensive hemorrhage and necrosis are uncommon. Characteristic tongue-like myometrial and lymphovascular invasion is usually present. The vascular architecture includes spiral arterioles, similar to those in ESNs and in the normal proliferative endometrium [4,9]. The cytomorphology also resembles proliferative endometrium, with sheets of small cells, round to oval nuclei and a scant cytoplasm. These cells usually whorl around spire arteriole-like vessels. Slow mitotic rate and an indolent clinical course are observed [8]. At the stromal level, LG-ESSs often retain a loose ECM resembling proliferative endometrium, but with focal deposition of collagens and proteoglycans that provide structural support for invasive growth. Increased MMP activity has been noted, which may facilitate stromal permeation and myometrial invasion [11].

### 2.3. Classification and Histopathology of High-Grade Endometrial Stromal Sarcomas (HG-ESSs)

HG-ESSs form a heterogeneous group of aggressive stromal-derived sarcomas. A variety of morphological and molecular characteristics is presented, depending on the genetic aberrations of each case [4]. On gross examination, a highly permeative mass, frequently polypoid, is observed. The presence of hemorrhage and necrosis is common. Tongue-like projections in the myometrium and lymphovascular invasion are also typical for these tumors [9]. Vascularity varies from delicate, thin-walled vessels to a strong, thick vascular architecture [4]. At the microscopic level, HG-ESSs lack the resemblance to proliferative endometrial stroma. The mass consists of two different cellular populations: high-grade round cells and low-grade spindle cells. The round cell component exhibits high cellularity. These cells are larger than normal stroma, with scant to moderate eosinophilic cytoplasm and irregular nuclei with evenly dispersed chromatin and absent prominent nucleoli. Cells are often organized into tight nest-like formations or pseudoglandular patterns. The mitotic index of this component is high. On the other hand, spindle cells present low or intermediate cellularity. There is a fibromyxoid morphology with ovoid to oblong nuclei, even chromatin and absent prominent nucleoli [8,9]. The mitotic index of this population is low. HG-ESSs show an aggressive clinical course with rapid progression and early recurrence [12].

### 2.4. Classification and Histopathology of Undifferentiated Uterine Sarcomas (UUSs)

UUSs is a diverse group that consists of uterine neoplasms lacking a specific differentiation line. By definition, this subgroup is diagnosed by exclusion. The gross appearance of these masses usually presents polypoid or intraluminal, large in size (>10 cm) and often accompanied by hemorrhage and extensive necrosis [8]. Destructive myometrial and lymphovascular invasion are common findings. UUS can be distinguished into monomorphic and pleomorphic subtypes. Microscopically, the morphology is not similar to that of the proliferative phase endometrium. These tumors are composed of epithelioid and/or spindled cells and exhibit high cytologic atypia [4]. Herringbone/storiform pattern or diffuse sheets are often observed. Cells with multiple, unconventional-shaped nuclei are frequently observed [8]. Mitotic activity is high, resulting in rapidly progressive clinical behavior. EST type histopathological characteristics are summarized in Table 1.

## 3. Immunohistochemical and Molecular Features

Endometrial stromal tumors share a common stromal origin but differ significantly in their underlying biology. Different subgroups of ESTs present characteristic patterns of protein expression and recurrent gene fusions, reflecting their unique molecular pathways and degrees of differentiation. Immunohistochemical and molecular profiling are crucial tools for the classification and treatment planning of these neoplasms.

### 3.1. Immunohistochemical and Molecular Features of Endometrial Stromal Nodules (ESNs)

This type of benign tumor shows a specific molecular and immunohistochemical profile. The most common chromosomal rearrangement among ESNs is JAZF1 SUZ12 gene fusion, presented in more than half of the cases [13]. Immunohistochemically, these tumors are CD10-positive, which is also the most widely used marker for EST diagnosis. Contrariwise, cyclin D1 staining is negative in this cancer subtype. Estrogen receptor alpha (ERα) and progesterone receptor (PR) are observed focally. Positive staining for smooth muscle actin (SMA), vimentin, and desmin is also noticed. WT1 and β-catenin are absent. P53 presents a wild-type expression [4].

### 3.2. Immunohistochemical and Molecular Features of Low-Grade Endometrial Stromal Sarcomas (LG-ESSs)

The majority of these tumors are affected by JAZF1 SUZ12 gene fusion. Other possible aberrations are JAZF1-PHF1, and the rare EPC1-PHF1, MEAF6-PHF1, ZC3H7-BCOR62 and MBTD1-CXorf67 [9]. LG-ESSs demonstrate the same immunohistochemical profile as ESNs. CD10 positivity is a typical feature and a useful marker for differentiating LG-ESS from HG-ESS. Strong ERα positivity, negative ERβ and normal PR staining have been reported. Androgen receptors are present in most cases. SMA and desmin are focally positive. WT1 is usually immunoreactive, while β-catenin is present in half of the cases.

### 3.3. Immunohistochemical and Molecular Features of High-Grade Endometrial Stromal Sarcomas (HG-ESSs)

This aggressive sub-group of tumors harbors YWHAE rearrangements and commonly carries the YWHAE-NUTM2 fusion. The immunohistochemical profile between the round and spindle cell populations that compose these masses is distinctive. In the round cell component, CD10 expression is typically low or absent, whereas cyclin D1 is overexpressed [4,9]. ER and PR show weak or negative staining, although positive staining has been recorded. The spindle-cell component presents a different expression pattern. CD10, ER and PR may show immunoreactivity. Cyclin D1 displays diffuse positivity. SMA and desmin are absent in both cell types. WT1 staining is low or negative, while p53 displays a wild-type pattern [8,9].

### 3.4. Immunohistochemical and Molecular Features of Undifferentiated Uterine Sarcomas (UUSs)

UUSs are diagnosed by exclusion, and thus the heterogeneity of this group precludes the existence of consistent molecular features [14]. It is characterized by complex karyotypes, and the absence of chromosomal translocations is usual. In rare UUS cases, JAZF1-SUZ12 fusion and YWHAE gene rearrangements have been reported [8]. The immunohistochemical profile also displays diversity. There is variable immunopositivity for CD10, ER, PR, SMA and desmin. Focal positivity for CD10 may be present [4]. Neoplasms composed of uniform cells tend to be ER- and PR-positive. P53 expression is usually aberrant in pleomorphic masses [8].

Immunohistochemical and molecular features of ESTs are summarized in Table 2.

## 4. Extracellular Matrix

### 4.1. Introduction

The TME of stromal sarcomas has been shown to play crucial roles in tumor cell signaling and function [15,16,17,18]. Gene expression profiling of stromal cells isolated from endometrial carcinoma cases revealed the expression of molecules involved in ECM interactions [19]. The endometrium is a dynamic tissue that undergoes cyclic remodeling through changes in the ECM, regulated by cyclic hormonal secretion, as well as cytokine and growth factor production in the endometrial microenvironment. The TME components include the cellular components and the ECM. The ECM comprises the non-cellular components of TME, secreted mainly by fibroblasts, that modulate cellular functions such as proliferation, migration, adhesion and apoptosis [20].

### 4.2. Cellular Components of Endometrial Cancer Microenvironment

Immune cells in the TME of sarcomas include innate immune cells such as neutrophils, tumor-associated macrophages (TAMs), tumor-associated dendritic cells (TADCs), and natural killer (NK) cells, as well as adaptive immune cells such as B cells and T cells. TAMs, TANs, and TADCs exhibit protumoral functions that drive metastasis, cell invasion, ECM remodeling, and angiogenesis by suppressing immune surveillance, thereby undermining antitumoral effects. Macrophage concentration is higher in endometrial cancer tissue than the physiological endometrium [21]. As the EST becomes malignant, TAMs are predominantly of the M2 type (pro-tumoral) [22] and express higher CD68+ levels than in the benign endometrium [21]. TAMs regulate as well the secretion of colony-stimulating factor 1 (CSF-1), tumor necrosis factor-alpha (TNF-a), interleukin 1 beta (IL-1b), interleukin 6 (IL-6), or SDF-1alpha and its receptor CXCR4 [23]. TADCs lose the antigen-presenting cell (APC) function and obtain protumoral effect [24].

More importantly, carcinogenesis is thought to begin with the differentiation of carcinoma-associated fibroblasts (CAF) [25]. These fibroblasts, found in the neoplasm, have increased growth capacity and enhanced expression of collagen and alpha-smooth muscle actin (αSMA). Higher levels of collagen I and III secreted by CAFs enable desmoplasia and formation of a dense collagen matrix [15]. Also, growth factors such as transforming growth factor beta (TGF-β) promote CAF formation. Furthermore, hepatocyte growth factor (HGF) produced by CAFs binds to its receptor on endometrial tumor cells, thereby increasing invasion capacity [26,27]. The chemokine SDF-1 binds to its receptor, CXCR4, modulating the migration of normal and malignant cells [28] and, thereafter, the invasion and metastasis of endometrial cancer cells [29]. Other produced cytokines in the endometrial tumor environment include IL-6, IL-8, monocyte chemotactic protein-1 (MCP-1 or CCL2), chemokine ligand 5 (CCL5 or RANTES) and vascular endothelial growth factor (VEGF) [30]. CAFs and TAMs modulate through SDF-1, TGF-β or HGF secretion the epithelial-to-mesenchymal transition (EMT), a process by which epithelial cells lose their characteristics and gain migratory and invasive properties, and/or the formation of a tumor-type microenvironment [31].

In addition, endothelial cells also play an essential role in cancer development, producing several factors that induce angiogenesis, such as vascular endothelial growth factor (VEGF), basic fibroblast growth factor (bFGF), IL-1beta, and TNF-alpha [32,33,34]. In gynecological tumor cells, perivascular tumor cells were shown to acquire a specific pericytic phenotype, enabling EMT [35].

### 4.3. ECM of Endometrial Cancer

The ECM is composed of collagens, enzymes, proteoglycans (PGs), glycosaminoglycans (GAGs), glycoproteins, extracellular phosphatases, kinases, cytokines, and growth factors. Its dynamic role within the TME is supported by evidence that it can sense and respond to mechanical cues, a process termed mechanosensing and mechanotransduction [15]. These mechanisms involve ECM-mediated signaling through adhesion molecules such as integrins, collagen, as well as mechanosensitive ion channels. The resulting downstream signaling activates cascades including the Rho/ROCK, MAPK, YAP/TAZ [36,37] and PI3K/Akt pathways, accompanied by alterations in cytoskeletal organization and gene transcription. Such regulation of cellular functions may, when dysregulated, contribute to pathologies such as cancer [15].

#### 4.3.1. GAGs

PGs consist of a core protein and one or more covalently attached sulfated GAG chains. Their multifunctional domains can interact with and modulate the function of a wide range of molecules, including chemokines, cytokines, growth factors, other ECM proteins, coagulation factors, and enzymes [15,31,38]. The type of GAGs incorporated into PGs are chondroitin sulfate (CS), dermatan sulfate (DS), heparan sulfate (HS) and keratan sulfate (KS). PGs’ GAG sulfation has been found to differ across tissue types, locations, and ages. Likewise, differences in GAG sulfation patterns, molecular weights, and structures can modify PG effects across various organs [39].

Notably, all the different GAG types are expressed in various compartments of the physiological endometrium and at different stages of the menstrual cycle [40,41,42,43,44]. CS is reported to be the primary sulfated GAG type found in the stromal cells of the endometrium [44]. Regarding endometrial carcinoma, Hosokawa et al. aimed to assess the localization of CS chains in the stromal region [45]. Indeed, in endometrial endometrioid carcinoma (EEC), CS was expressed in the endometrial cancerous stromal formed areas, supporting the tumor tissue structure. As the grade increased, the localization of CS in the ECC mirrored changes in stromal structure. In contrast, in endometrial serous carcinoma (ESC), CS localization was only partially independent of structural stromal changes [45]. Similar observations have been made when comparing normal and malignant human endometrium, where differential expression of PGs and GAGs, including CS and HS, was reported to correlate with malignant transformation and tumor progression [46].

Hyaluronan (HA), a free, secreted GAG, has been widely studied in cancer, with its effects mostly correlated with its molecular size [47,48,49]. Interestingly, HA has been linked to TAM differentiation toward the M2 phenotype in the tumor stroma [50]. Tumors with decreased HA synthesis presented lower infiltration rates of monocytes and TAMs [51]. In endometrial cancer, HA levels were elevated due to reduced hyaluronidase activity [52]. Moreover, receptor for hyaluronic acid-mediated motility (RHAMM), an HA receptor involved in cancer pathogenesis [53], was found to enhance the invasion and metastasis of endometrial carcinomas [54]. Consistent with these findings, high stromal HA content has been associated with poor outcomes in other gynecological malignancies such as ovarian and breast cancer [55,56]. Studies on the role of HA in EST-associated angiogenesis and progression are necessary to further understand its significance.

#### 4.3.2. PGs

PGs can be located either on the cell surface or secreted into the ECM. Examples of cell surface PGs include syndecans and glypicans. Syndecans are transmembrane proteins bearing HS side chains and have been shown to interact with and modulate the actions of various growth factors in the ECM. Glypicans are glycosylphosphatidylinositol-anchored PGs on the cell’s outer surface that also regulate multiple intracellular signaling pathways. The secreted into the ECM PGs include small leucine-rich PGs (SLRPs), aggrecan, versicans and basement membrane PGs. These conjugated proteins are involved in collagen fibrillogenesis, maintenance of osmotic pressure, presentation of soluble molecules to cells, cell structure, and angiogenesis [15,39,57]. PGs play essential roles in embryogenesis and organogenesis, and changes in their expression have been linked to tumor formation and cancer development [20]. Additional evidence supports their role in cancer cell adhesion, migration, and invasion.

The female reproductive organs, particularly the endometrium, undergo continuous physiological turnover and remodeling under the regulation of ovarian steroids and pituitary hormones. While studies, mainly in animal models, have provided insights into the role of PGs and their hormonal regulation in the reproductive system, data from human tissues and uterine pathologies remain limited and warrant further investigation [20,58,59,60].

SLRPs, which share repeats of leucine-rich structural motifs and cysteine residues, contribute to collagen fibrillogenesis and matrix assembly in the ECM [57,61]. Some of the SLRP family members, extensively studied, are fibromodulin, biglycan, decorin and lumican [62]. Their role and regulation by hormones of the reproductive system, especially for decorin and biglycan, have been described [59,60]. More importantly, SLRPs have been related to cancer cell functions such as proliferation and migration [57]. Decorin and lumican can suppress growth factor signaling (e.g., TGF-β and EGFR), while biglycan may promote inflammatory signaling through TLR2/4, underscoring their dual tumor-suppressive and tumor-promoting roles [63,64,65].

As far as benign tumors are concerned, such as uterine fibroids/leiomyomas, PGs’ actions have been studied, as fibroid formation can affect human embryo implantation, placentation, and fetal development [58]. Fibromodulin, decorin and biglycan can contribute to collagen fibrillogenesis. Fibromodulin is highly expressed in the uterine fibroid tissue, at the proliferative phase of the menstrual cycle [66]. Decorin, versican, syndecan-4 and ECM protein-2 were also overexpressed in fibroid tissue compared to the normal myometrium [67]. Furthermore, decorin in the uterine fibroid exhibits a unique structure with longer GAG side chains [68,69]. Biglycan was associated with thick collagen fibril formation in decidualized cells of the endometrium, whereas decorin was related, in contrast, with thin collagen fibrils in non-decidualized endometrial areas [70]. Notably, biglycan has been suggested to modulate collagen fibrillogenesis in the absence of decorin. This compensatory mechanism has also been confirmed in uterine fibroids, where biglycan supports abnormal collagen accumulation and stiffening [37]. Decorin depleted mice show enhanced biglycan expression in the non-decidualized endometrium together with an irregular profile of fibrils [71].

PGs’ involvement specifically in EST formation is limited. Lucariello et al. (2015) studied the distribution of SLRPs in the normal endometrium and their changes during pathogenesis, such as hyperplasia [59]. Expression of biglycan and lumican was low in polyps and during hyperplasia, compared to the physiological endometrium, whereas fibromodulin and decorin were absent [59]. Other studies have shown abnormal expression of versican and syndecans in endometrial hyperplasia and carcinoma, correlating with enhanced proliferation, invasion, angiogenesis, and altered ECM remodeling, suggesting that PG profiles could serve as biomarkers of early pathological changes [72,73,74,75].

The above observations on fibroid formation by ECM molecules, together with how shifts in PG expression (SLRPs, versican, and syndecans) can affect endometrial hyperplasia and tumor formation, suggest that dysregulated PG networks may represent an early step linking benign remodeling to malignant EST transformation. The latter needs to be verified in future studies. Taken together, the dynamic regulation of PGs in normal endometrium, fibroids, hyperplasia, and carcinoma underscores their potential as diagnostic and prognostic biomarkers and as promising therapeutic targets in uterine stroma–derived pathologies. An overview of PGs expression in uterine tissues and tumors is presented in Table 3.

#### 4.3.3. Osteopontin (OPN)

Osteopontin (OPN), a 70 kDa particle of phosphorylated N-linked glycoprotein, was first discovered in bone ECM. It binds to the CD44 receptor and can modulate cell signaling pathways by interacting with integrins (αvβ3 and αvβ5) and several other molecules [76,77,78]. OPN has the ability to bind osteocalcin, type I collagen, and fibronectin [79] and is involved in cell functions such as adhesion, tumorigenesis, angiogenesis, and metastasis, and may also serve as an independent prognostic biomarker in a variety of cancers [68,69]. Its overexpression has been associated with several tumor types and linked to signaling pathways such as ERK1/2, PI3K/AKT, nuclear factor kappa-light-chain enhancer of activated B cells (NF-κB), and MMP-2 expression, modulating cell apoptosis, migration, and proliferation [67,79].

Studies have described OPN’s action in the uterus and placenta during the menstrual cycle and pregnancy [80]. OPN is thought to participate and is overexpressed in endometriosis, a benign condition with characteristics similar to those of malignant tumors, and in adenomyosis [81,82]. Notably, OPN expression can also be influenced by progesterone, and its binding partner, β3 integrin, was found to increase in response to epidermal growth factor or heparin-binding epidermal growth factor.

Notably, OPN can modulate the TME by regulating immune cells, altering interleukin and interferon-γ secretion [83,84,85,86]. Indeed, OPN has been associated with head, neck, lungs, breast, liver, stomach, colon, cervical, ovarian and endometrial cancer [87,88]. Another essential fact about cancer development is that OPN has been shown to enhance EMT, thereby aiding cancer metastasis and resistance to therapies [87,89].

OPN’s role in endometrial cancer has been correlated with disease progression, patient survival, cell invasion, cell migration and EMT [83,88,90,91]. Al-Maghrabi et al. (2020) investigated the relationship between OPN immunostaining and clinicopathological features in 71 endometrial carcinoma tissue samples [92]. They showed that increased OPN immunostaining, mainly on granular epithelial cells, was more frequent in non-neoplastic tissues than in endometrial carcinoma [92]. Indeed, enhanced OPN immunostaining was reported in 43.6% of low-grade endometrial cancer and in 21.1% of high-grade endometrial cancer [92]. There is controversy among studies regarding whether OPN expression relates to EC grade or EC tumor size [92]. Studies to date do not specifically report OPN’s expression in endometrial stromal cells compared to other cell types. Given OPN’s well-established role in modulating stromal cell function in tumorigenesis and metastasis [93], future studies should aim to describe osteopontin-stromal-mediated pathways in EST cancer development and behavior.

#### 4.3.4. Collagens

Collagens are large, rigid molecules of the ECM that regulate the mechanical strength of tissues in the body. Twenty-nine types of collagens, encoded by numerous genes, are abundantly expressed in all tissues [94]. Tumors, which are characterized by increased collagen synthesis, are more aggressive and rich in blood vessels, thus suggesting that collagen instead promotes invasiveness and angiogenesis [95]. Fibroids, benign tumors, are characterized by proliferation and persistence of cells due to resistance to apoptosis, as well as increased secretion of collagens, deposition of abundant, highly cross-linked, and disoriented collagen fibrils, and secretion of proteoglycans and other matrix components [96]. In uterine fibroids, types I, III, and V collagens are expressed, with fibrils that exhibit a disordered pattern that is increased in amount compared to the adjacent myometrium. Collagen has been found to colocalize with decorin in both the myometrium and fibroids, suggesting that this interaction may be necessary for the uterus [69,97,98]. Other important ECM molecules in uterine fibroids are hyalectans (molecules that interact with hyaluronan), elastin [99,100], fibronectin and integrins (transmembrane receptors) [101,102,103,104]. Collagen-binding integrins of the β1 subfamily are expressed in fibroids as well as in the myometrium and in the cervix [105,106,107], where they regulate cytoskeletal integrity and growth [107]. Furthermore, collagen fibrils in uterine fibroids have smaller lengths and are disoriented compared to normal myometrium Automatic citation updates are disabled. To see the bibliography, click Refresh in the Zotero tab. [98,108]. Other ECM components of fibroids include different percentages of GAG CS and DS compared with the normal myometrium [109].

Collagen cross-linking enzymes such as lysyl oxidase (LOX) are also upregulated, enhancing ECM stiffness and supporting fibroid progression [110]. In endometrial carcinoma, overexpression of type I and type III collagens has been associated with tumor invasion, metastasis, and poor prognosis [111,112], suggesting that collagen remodeling is not only a hallmark of benign fibroids but also contributes to malignant stromal transformation.

#### 4.3.5. MMPs-TIMPs

ECM proteolysis events are also crucial and influence cellular changes within each organ. Especially in the endometrial tissue, ECM synthesis and degradation are essential throughout the menstrual cycle for optimal function [113]. Matrix metalloproteinases (MMPs) and their natural tissue inhibitors of metalloproteinases (TIMPs) act synergistically to regulate ECM turnover. MMPs and TIMPs levels are involved in several key aspects of tumor growth, invasion, and metastasis [4,114]. The actions of MMPs and TIMPs have been studied and partially described in benign human reproductive tissues; further knowledge is necessary in pathological states such as cancer [113].

Thus, overexpression of MMP types 1, 2, 7, and 9 has been reported in endometrial cancer, and it was associated with poor prognosis [11,115,116,117,118]. Specifically, in endometrial stromal sarcomas, MMP 2,3, and 9 have been studied. MMP 3 and 9 are expressed more diffusely in high-grade cases, and MMP2 presents higher expression in HG-ESS [119]. Likewise, MMP14, a membrane-bound MMP, has been correlated with increased myometrial and lymph node invasion [120]. Gomez-Macias et al. (2018) showed that increased expression of MMP11 may be used as a prognostic marker in patients with endometrioid cancer [121]. Indeed, MMP11 is secreted in its active form, unlike other MMPs, which are first expressed as proenzymes; thus, MMP11 may have a unique function in tumor development, although it does not degrade major ECM components [122,123,124,125]. In a more recent study, the expression of MMP11 and MMP17 in uterine corpus endometrial carcinoma (UCEC) was determined, and bioinformatics tools were used to correlate their expression with patient prognosis, as they influence immune cell infiltration [126]. A total of 22 MMPs were found to be abnormally expressed in UCEC tumor tissues, with MMP11 and MMP17 significantly enriched in the tumor’s ECM [126]. They were associated with pathways involving ECM degradation, glycolytic metabolism, and PI3K-Akt signaling [126]. Moreover, infiltration of natural killer (NK), mast, and NK CD56 bright cells was enhanced in tumor tissues with high MMP11 and MMP17 expression [126]. Moreover, in uterine leiomyosarcoma, increased expression of MMP2 and MMP9 has been associated with enhanced invasiveness and poorer clinical outcome, further underscoring the prognostic significance of MMP-driven ECM remodeling in uterine mesenchymal tumors [11].

Emerging data also suggest that an imbalance between MMPs and TIMPs influences therapeutic resistance, since high MMP activity may increase ECM stiffness and promote an invasive niche, while elevated TIMP expression can paradoxically support tumor growth and angiogenesis [127,128]. These findings highlight the dual role of MMP/TIMP systems as both biomarkers and potential therapeutic targets in endometrial cancers.

#### 4.3.6. Other ECM Molecules

Legumain (LGMN), a cysteine endopeptidase present in the ECM, and Nidogen, a basement membrane glycoprotein that acts as a bridge between the ECM molecules (laminin-1 and type IV collagen) and binds to integrins, were presented to associate with increased migration and invasion of endometrial tumor cells in vitro [129,130]. The latter identified novel ECM molecules, whose expression may have significant, as yet unknown roles in endometrial pathologies. Further studies are needed to understand the significance of NID1 and LGMN in the development of ESTs.

**Figure 1:**
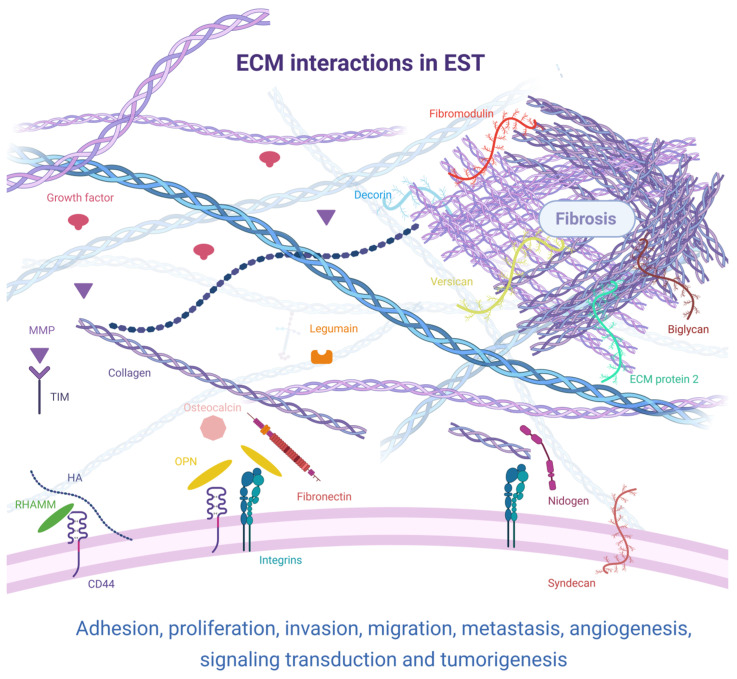
ECM interactions in endometrial stromal tumors (ESTs). In the EST, several key-molecules have been found to regulate tumor formation and cancer development. Hyaluronan (HA) binding to its receptor, hyaluronic acid-mediated motility (RHAMM), enhances invasion and metastasis of endometrial carcinomas. Osteopontin (OPN) interacts with CD44 receptor, osteocalcin, type I collagen, and fibronectin, changing adhesion, tumorigenesis, angiogenesis, and metastasis. Nidogen connects to ECM molecules (like collagen) and binds to integrins, resulting in increased migration and invasion. In addition, proteoglycans and their association with collagen, crucial for fibrillogenesis, are presented (decorin, fibromodulin, biglycan, ECM protein-2, syndecans and versican). MMP enzymes and their inhibitors TIMPs are essential for ECM remodeling, cancer progression and metastasis. Created in BioRender. Nikitovic, D. (2025) https://BioRender.com/k0uuomf (accessed on 23 September 2025).

Figure 1 summarizes the existing knowledge in ECM molecules’ interaction present in ESTs.

## 5. Clinical Presentation and Diagnostic Approach

The clinical presentation of the uterine sarcomas is non-specific. It ranges from being asymptomatic to symptoms associated with pelvic pathology like vaginal bleeding, palpable pelvic mass, and pelvic pain, due to potential pressure effects on adjacent organs or local nerve involvement [131]. In case of extra-uterine disease, symptoms may include abdominal pain due to peritoneal spread affecting bowel function and abdominal distension in case of development of malignant ascites [132].

The most accepted initial imaging approach is, by far, transvaginal ultrasound, with the abdominal approach reserved for cases suspicious of advanced disease. The ultrasound imaging techniques to be applied are high-resolution grayscale imaging combined with color Doppler to assess the endometrium and myometrium [132] thoroughly. The standard imaging is the traditional two-dimensional, with the three-dimensional used for offline evaluation and referral to experts for a second opinion [132]. The ultrasound findings that may lead to a uterine sarcoma diagnosis comprise the existence of a solid heterogeneous mass, often with cystic degenerative appearance, with infiltrative, ambiguous borders suggestive of myometrial infiltration [133]. Sometimes the image refers to disease nodules within the myometrium. As expected, due to increased angiogenesis, these tumors are densely vascularized, a fact well-depicted by Doppler sonography [133].

Suspicious ultrasound findings are followed by MRI, the second step in the diagnostic approach. So far, several protocols have been proposed to differentiate uterine leiomyosarcoma from leiomyomas [134,135]. In the recent ESGO/EURACAN/GCIG guidelines, an array of eight features, based mainly on pathologic diffusion-weighted imaging, along with specific pre-contrast and post-contrast findings, may gradually increase the likelihood of uterine sarcoma (Table 3). A high suspicion for sarcoma arises when four out of eight imaging features are identified [132]. In cases of indeterminate masses, 18F-FDG PET/CT is considered a valuable tool [136]. It should be noted that several benign fibroids may show elevated 18F-FDG uptake, leading to false-negative conclusions. This may be attributed to either cellular myomas or myomas with prominent vascularization [137,138].

Histological sampling should be performed wherever possible. In case of endometrial stromal sarcoma, sampling can be performed either by dilatation and curettage (D&C) or by hysteroscopy under direct vision, since ESS is usually accessible via the uterine cavity. In a different case, pre-operative biopsy is not the standard of care choice [132]. However, in the rare case of an unavailable hysterectomy, a core biopsy is considered an option. Core biopsy can be performed either trans-abdominally or via the endometrial cavity. A transabdominal biopsy could also be performed when peritoneal disease is identified. The risk of needle-tract seeding is negligible [139]. Due to the rarity of uterine sarcomas, the histological sample should be sent to expert pathologists for microscopy and genomic analysis.

All patients diagnosed with uterine sarcoma, including those receiving incomplete tumor resection or those with incidental findings of uterine sarcoma, should be offered a chest/abdomen/pelvis contrast-enhanced CT scan, or an abdomen/pelvis MRI followed by a chest CT to complete proper staging and be referred for further management [132]. The International Federation of Gynecology and Obstetrics (FIGO) staging for uterine leiomyosarcoma and endometrial stromal sarcomas is presented in Table 4 [140].

## 6. Treatment Options

### 6.1. Surgical Treatment

Surgery is considered the mainstream treatment of uterine sarcomas. Although, in general, minimally invasive techniques are effective in delivering surgical treatment, the small risk of intraperitoneal uterine rupture should be taken into consideration, especially since it is an independent negative prognosticator [141]. Minimally invasive surgical treatment should be offered only in cases where uterine integrity can be assured. In the same direction, in cases of high clinical suspicion for uterine sarcoma and the possibility of electromechanical specimen morcellation, minimally invasive techniques should be avoided. Pre-operative imaging is neither specific nor sensitive enough to exclude uterine sarcomas efficiently. In that view, the risk of sarcoma is estimated by clinical criteria, namely, peri-or menopausal state, fast-growing or new myoma, abdominal discomfort, pelvic pain or vaginal bleeding, and tamoxifen administration. All these conditions dictate avoiding specimen morcellation and the concurrent use of abdominal (open) surgery.

Benign or premalignant pathology. In case of endometrial stromal nodule or smooth muscle tumor of unknown malignant potential (STUMP), total hysterectomy with bilateral salpingo-oophorectomy is the standard and definitive treatment [142,143]. In pre-menopausal women, salpingo-oophorectomy can be omitted.

Early-stage uterine sarcomas (FIGO I and II). The mainstream surgical treatment of uterine sarcomas confined to the uterus is total abdominal hysterectomy with bilateral salpingo-oophorectomy [144]. Its application is standard for peri- and menopausal patients, but currently controversial for women with reproductive potential, in terms of ovarian preservation. Two meta-analyses have shown that oophorectomy does not decrease overall survival [145,146]. Even in patients with ER/PR-positive uterine leiomyosarcoma, oophorectomy did not improve survival [147]. In high-grade ESS cases, ER/PR is commonly absent, and thus ovarian preservation is not anticipated to affect survival parameters. In line with the evidence, the current guidelines suggest that oophorectomy, in pre-menopausal women with any sarcoma, should be restricted only in cases with ovarian involvement [132]. The role of lymphadenectomy has not yet been delineated. Current guidelines do not recommend systematic lymphadenectomy as part of contemporary practice; selective lymph node dissection is recommended only in cases of enlarged lymph nodes found intraoperatively [132]. However, a recent meta-analysis of 26,693 patients showed that patients diagnosed with high-grade ESS may benefit in terms of overall survival, while patients with uterine leiomyosarcoma or low-grade ESS may not [148]. This is partly in line with a previous meta-analysis in 6412 patients with apparent early-stage uterine sarcoma; lymphadenectomy was significantly associated with improved survival only in case of high-grade endometrial sarcoma or undifferentiated sarcoma [149]. These meta-analyses may indicate a role for lymphadenectomy in selected cases based on initial histology.

Advanced-stage uterine sarcomas (FIGO III and IV). Advanced uterine sarcomas are treated surgically in a fashion similar to ovarian cancer, since an important prognostic factor is the volume of residual tumor after debulking surgery. In that view, primary cytoreduction may involve total abdominal hysterectomy with bilateral salpingo-oophorectomy, omentectomy, peritonectomy, small- or large-bowel resections, and splenectomy [144]. Lymphadenectomy is limited to enlarged intraoperative lymph nodes. In accordance with treating advanced uterine sarcomas as advanced ovarian cancer, hyperthermic intraperitoneal chemotherapy (HIPEC) was also tested without significant results. The mortality rate was noted 4% [150]. For stage IV, oligometastatic disease can be a candidate for a surgical approach, provided that complete tumor resection is feasible with acceptable operative morbidity. Alternatively, neoadjuvant chemotherapy or hormonal treatment (in case of low-grade ESS) is also currently recommended, in the aim of achieving resectable disease [2,132].

Recurrent disease. Disease recurrence is usually located in the abdomen/pelvis or as lung metastases. The evidence for treating recurrent uterine sarcoma with secondary cytoreduction is currently weak. As a result, it is recommended that decisions be made under a case-by-case scheme. This decision depends on tumor histology, previous type of surgery, efficacy of oncology cytoreduction (complete or not) and prior chemotherapy or hormonal treatment schemes [132]. Since cytoreduction is expected to reach its maximal extent, the patient’s general condition and tumor resectability should also be considered. In this context, pre-operative chemotherapy (2–4 cycles), hormonal treatment, and, in specific cases, local ablation with radiotherapy or radiofrequency could be offered. The aim here is to minimize tumor burden, facilitating complete recurrence removal with less morbidity [151] In case of low-grade ESS with previous preservation of the ovaries, bilateral oophorectomy is offered to minimize the estrogen effect on the tumor [144]. Alternatively, gonadotropin-releasing hormone analogs should be considered [152].

Residual disease. In terms of residual disease, either due to subtotal hysterectomy or due to incidental finding of sarcoma after myomectomy, completion surgery should be performed. In the case of a subtotal hysterectomy, completion surgery refers to cervical stump removal. In case of total hysterectomy, completion surgery concerns myomectomy followed by bilateral salpingo-oophorectomy, usually in menopausal women [132].

### 6.2. Adjuvant/Neo-Adjuvant Treatment

#### 6.2.1. Uterine Leiomyosarcoma

Localized uterine leiomyosarcoma (FIGO I). Although limited, the current evidence suggests no survival benefit with adjuvant chemotherapy or radiotherapy [153,154,155]. In that view, surveillance is the gold standard. Due to a lack of robust evidence, any alternative approach should be discussed appropriately with the patient and, preferably, offered in the context of a research protocol.

Locally advanced uterine leiomyosarcoma (FIGO II–III). Adjuvant chemotherapy can be considered in case of locally advanced uterine leiomyosarcoma, since it contributes significantly to overall survival [156]. The chemotherapy schemes include single-agent doxorubicin. The gemcitabine-docetaxel combination can be considered an alternative. It has been shown that these schemes do not differ significantly in terms of PFS. However, the second scheme was associated with more frequent and more severe adverse effects [157]. In that view, single-agent doxorubicin remains the first option, with the combination gemcitabine-docetaxel to be employed in case of doxorubicin intolerance. Doxorubicin-based schemes are also used in FIGO stage III as definitive or pre-operative chemotherapy, aiming to improve tumor resectability. To this end, combinations of doxorubicin with trabectedin or dacarbazine have been suggested as effective treatment [158,159]. Radiotherapy may also be offered as adjuvant treatment to reduce the risk of pelvic relapse [132].

Metastatic uterine leiomyosarcoma (FIGO IV). In metastatic disease, chemotherapy is the mainstream option. The first-line chemotherapy schemes are both doxorubicin-based combinations and non-doxorubicin-based: doxorubicin-trabectedin, doxorubicin-dacarbazine, gemcitabine-docetaxel, single-agent doxorubicin, single-agent liposomal doxorubicin, and single-agent gemcitabine [132,160]. Second-line chemotherapy decision-making depends primarily on prior regimens and the patient’s response. Also, existing and potential cytotoxicity, as well as the patient’s performance status, should be considered. These therapeutic schemes do not differ from the first-line options, with trabectedin, dacarbazine, and pazopanib as single-agent options that have shown promising outcomes [161]. A recent meta-analysis demonstrates that the well-established gemcitabine-docetaxel regimen provides the most significant benefit when used as second-line treatment [162]. Low-grade metastatic disease. This group of patients depicts a rare case of slowly progressive uterine leiomyosarcoma initially diagnosed as STUMP. Progression, although indolent, may include abdominal or lung metastases. Positive hormonal receptor expression is usually managed with hormonal treatment [163].

#### 6.2.2. Low-Grade Endometrial Stromal Sarcoma (LG-ESS)

Low-grade ESS typically expresses hormone receptors, which justifies the use of hormonal treatment. Anti-estrogenic treatment so far includes aromatase inhibitors, progestins, GnRH analogs, and estrogen receptor degraders like fulvestrant (Table 5). A series of retrospective studies supports aromatase inhibitors as superior, with greater efficacy and a lower rate of adverse effects. The use of tamoxifen, a selective estrogen receptor modulator, is widely used as hormonal treatment in hormone receptor-positive breast cancer cases. However, its use in ESS is contraindicated since tamoxifen acts as an estrogen receptor agonist in the uterus. There is no clear recommendation so far regarding treatment duration [144]. In accordance with breast cancer treatments, endocrine treatment is administered for at least 2 years. It is unclear whether treatment should be lifelong or discontinued upon diagnosis of recurrence [132].

Endocrine therapy is not currently recommended in FIGO stage I disease, provided that surgical treatment is commonly adequate. On the contrary, FIGO stage I is treated with morcellation (increasing the risk for local tumor dissemination) and all the other FIGO stages are considered eligible for endocrine therapy [144]. Endocrine treatment is also recommended in the case of fertility preservation with uterine preservation. Such an approach, referring to local low-grade ESS excision without hysterectomy, may offer an opportunity for reproduction. However, there is a significant cost of regional or distant recurrence and mortality [164,165]. The evidence supporting fertility preservation is relatively weak, yet it reveals a satisfactory rate of successful pregnancies [166]. Endocrine treatment is thought to be offered up to the initiation of an attempt to conceive (either via assisted reproduction techniques or not).

Endocrine therapy is also the primary treatment in case of recurrence or metastatic disease. First-line anti-estrogenic interventions usually succeed in disease response. This further supports the concept of debulking surgery after 3 months of treatment as sound. In case of tumor response, the residual nodules are also eligible for secondary debulking surgery [2]. Second-line treatment is based on the same pharmaceutical schemes, taking into account the previous agents administered along with their impact on disease progression. The central notion is selecting anti-estrogenic agents beyond those offered in first-line treatment. In that view, fulvestrant, an endocrine receptor degrader, is a reasonable choice for second-line treatment [167].

In case of resistance to multiple anti-estrogenic schemes, chemotherapy is considered the last alternative. The therapeutic schemes do not differ from the central concept of doxorubicin-based treatment, with the alternative option being the combination of gemcitabine and docetaxel. The efficacy of chemotherapy in low-grade ESS has not been evaluated to date. This is mainly attributed to the lack of proper classification over the last decades. Before the establishment of endocrine treatment, most reports included all ESS cases without adequate grouping into low- or high-grade disease. The current trend is evaluating the efficacy of CDK4/6 or PI3KCA inhibitors as add-ons to aromatase inhibitors. The perception of this approach stems from the evidence accumulated from breast cancer treatment. Results from relevant studies can clarify whether such a scheme is also effective in low-grade ESS. Chemotherapy may also be considered in cases of de novo ESR1 hotspot mutations, which are usually linked to aromatase inhibitor resistance and transformation from low- to high-grade ESS [132,168]. The fact that aromatase inhibitor resistance differs from estrogen receptor degrader resistance makes the latter a promising option. Thus, if ESR1 mutations have been demonstrated, fulvestrant is considered the treatment of choice [132].

Radiotherapy is not suggested as adjuvant treatment, especially in localized disease. Although there is weak evidence about a possible role in local control in locoregional disease, no significant impact on survival has been shown so far. Finally, radiotherapy may be offered as a palliative intervention.

#### 6.2.3. High-Grade Endometrial Stroma Sarcoma/Undifferentiated Uterine Sarcoma

High-grade ESS and UUS are entities characterized by aggressive behavior, with significant risk for recurrence or metastasis. Aggressive behavior implies systemic treatment to be the only option in the aim of reducing recurrence and metastatic rates. However, the evidence for properly operated FIGO stage I disease is not as supportive as anticipated. Despite no recommendation supporting chemotherapy for FIGO stage I patients, it is offered in several centers after thorough discussion, both at the multidisciplinary team meeting and with the patient [132]. On the contrary, at FIGO stages II–IV, chemotherapy is the gold standard either as a neoadjuvant or adjuvant approach [169,170]. Therapeutic schemes are still doxorubicin-based either as a single agent or combined with ifosfamide [171]. A reasonable alternative option is the combination gemcitabine-docetaxel. In case of second line treatment, single agent ifosfamide, gemcitabine, trabectedin, pazopanib and doxorubicin may be administered.

All FIGO stages for uterine leiomyosarcomas and endometrial stromal sarcomas are summarized in Table 6.

Radiotherapy may be used for local control in recurrent or metastatic disease [155]. It may also be considered in a pre-operative or post-operative treatment setup.

Targeted therapies are considered the future in treating high-grade ESS. A logical approach would require focus on the molecular characteristics of this category of tumors, namely the YWHAE::NUMT2A/B gene fusion, the BCOR gene rearrangements, and the c-kit overexpression, along with their downstream pathways [172]. Promising results may also stem from anti-angiogenetic treatment and immunotherapy (Table 7).

## 7. Prognosis

An endometrial stromal nodule is a benign finding with excellent prognosis. No further follow-up is needed. In case of STUMP, despite the fact that no further treatment is recommended, there is a risk for relapse that may reach up to 36.4% [143,177]. Despite the moderate 5-year disease-free survival (66%), STUMP patients have a very good prognosis (92–100% 5-year overall survival) [177]. In case of recurrence, this may be identified either as STUMP or as leiomyosarcoma. Possible sites for recurrence are pelvis, ovary, abdomen, omentum, retroperitoneum, liver, lung, pleura, bone, brain and spine. Surgical resection, when feasible, is the gold standard approach in treating recurrences.

**Figure 2:**
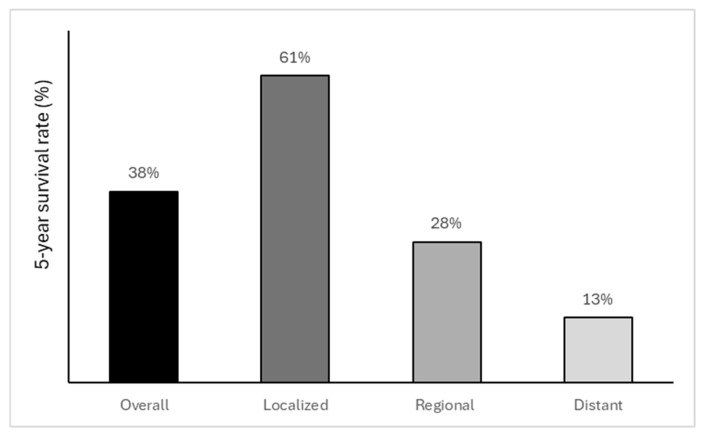
Five-year survival rate of leiomyosarcoma. American Cancer Society. Uterine Sarcoma Early Detection Diagnosis, and Staging https://www.cancer.org/cancer/types/uterine-sarcoma/detection-diagnosis-staging/survival-rates.html (accessed on 24 October 2025).

According to the American Cancer Society, based on SEER data, leiomyosarcoma survival depends on the stage at diagnosis and the mitotic index [143,178]. In localized disease, the 5-year relative survival rate is 61%, whereas in regional and distant disease, it is 28% and 13%, respectively (Figure 2). The overall survival rate is 38% [179] (Figure 2). Uterine leiomyosarcomas seem to be rather aggressive, with a high rate of recurrence even in cases of early-stage disease. Their recurrences may be either pelvic/abdominal or distant, usually involving the lungs [143]. Due to the increased risk of relapse, patients are under close follow-up.

Low-grade ESS presents an indolent progression. Survival rates are very good for FIGO stages I–II, reaching up to 90%; however, in advanced stages, survival is low to moderate (approximately 50%) [5]. Low-grade ESS is expected to recur even in early-stage disease at a later time point, with recurrences located in the abdomen/pelvis or distantly in the lungs [180]. Hormonal receptor expression is considered a favorable prognostic factor, as these cases usually respond well to hormonal therapy. Due to the significant risk for recurrence, patients continue to be under long-term surveillance.

High-grade ESS are considered very aggressive tumors with a significant risk of recurrence or distant metastasis, even in the case of early diagnosis. The major prognosticators have been reported to be the initial FIGO stage at time of diagnosis, tumor size, minimum and average values of CA125, menopause, history of uterine leiomyoma, and endometriosis [181]. The presence of specific gene fusions (ZC3H7BBCOR, EPC1-SUZ12 and EPC1-BCOR) has also been associated with poorer prognosis [2].

Finally, the role of surgical efficacy in cytoreduction has been recently highlighted in a multicenter retrospective study [182]. By reviewing 683 patients with all types of sarcoma, the authors have highlighted incomplete cytoreduction, tumor persistence, FIGO stage, extrauterine involvement, and tumor margin involvement as the most relevant factors affecting overall survival in uterine sarcoma.

Alterations in the ECM have been observed in uterine sarcomas and correlate with aggressive phenotypes [183]. Moreover, reduced ECM expression has been associated with a favorable prognosis and diminished YAP activation in uterine sarcomas [184]. Consistently, large-scale analyses of soft tissue sarcomas demonstrate that distinct ECM network states are associated with survival outcomes [185]. Nevertheless, a detailed assessment of the expression patterns and prognostic value of markers specific to EST is lacking. Possible candidates suggested are HA receptors RHAMM and CD44 [186,187,188]. RHAMM expression may serve as a prognostic biomarker in uterine carcinosarcoma, according to a bioinformatics analysis [186]. Expression of CD44 standard and isoforms v3 and v6 in uterine smooth muscle tumors was found to be a possible diagnostic tool for leiomyosarcoma [189]. In contrast, CD44v6 membranous staining was reported to help differentiate malignant from benign endometrial tissue, but it was not associated with endometrial cancer staging or prognosis [190]. In addition, Syndecan 1, a transmembrane PG, was described as a tool for assessing malignancy risk [191]. Biglycan, like other matrix proteins, may also serve as a prognostic marker due to its role in the progression of human endometrial cancer [192]. Moreover, MMP11 and MMP17 were suggested as potential biomarkers for the prognosis of uterine corpus endometrial carcinoma [126]. Cho et al., showed that in FIGO stage I endometrial cancer, osteopontin correctly identified the majority of cases (62.1%) and pinpointed osteopontin as an independent prognostic factor for disease-free survival [88].

## 8. Emerging Therapies and Future Directions

Over the last decades, the perception of cancer as a simple mass of uncontrollable proliferation has changed. Modern oncology recognizes tumors as complex entities with a deep genetic, epigenetic, and biochemical background. This transition is directly reflected in the emerging therapeutic approaches. Studies focused on targeted molecular therapies, such as receptor tyrosine kinase inhibitors and epigenetic modulators, the tumor microenvironment, and personalized medicine are gradually improving or replacing current aggressive treatments, such as chemotherapy and actinotherapy.

### 8.1. Tumors’ Microenvironment and ECM-Targeted Therapies

Studies on the modulation of ECM molecules and their interactions with the TME’s cellular components in ESTs primarily aim to discover new target molecules and/or mechanisms for future therapeutic strategies. ECM molecules have already been described as candidate molecules for different cancer therapy strategies [38,193]. However, only a few such candidates have been identified to date, as studies specifically addressing the ECM in ESTs remain scarce. OPN-mediated mechanisms, reviewed recently by [194], represent a promising avenue for EST therapy. OPN inhibitors were shown to block proliferation, invasion, immune invasiveness, radiotherapy resistance, and genes involved in metastatic capabilities. Moreover, OPN can remodel the ECM of ESTs by altering the expression of MMPs, CD44, and integrins—molecules central to EMT, invasion, and metastasis [77,78,85,86,89,195,196]. Beyond OPN, modulation of PGs has been investigated in the context of endometrial fibrosis, with translational implications for stromal tumors. For example, treatment with gonadotropin-releasing hormone analogs reduced fibromodulin expression in fibroid tissues to physiological levels, while all-trans retinoic acid suppressed leiomyoma cell proliferation by downregulating versican [58]. Another approach in fibroids involves the use of collagenase, which degrades collagens and decreases TME stiffness [197]. In addition, PGs such as decorin and biglycan—both implicated in fibril formation and matrix organization of endometrial tumors—may also serve as future therapeutic targets or modulators of disease progression [57,64].

Furthermore, selective inhibitory antibodies targeting angiogenesis-promoting MMPs have been studied across different cancer types, demonstrating clinically favorable outcomes [198,199]. MMP-14 (MT1-MMP) has also been suggested as a great candidate for anti-cancer treatment, due to the significant involvement in migration and invasion. However, the clinical application of MMP-14 inhibitors is hindered by significant off-target side effects. The use of more specific, small-molecule inhibitors constitutes a promising future approach [200]. In terms of endometrial cancer, CGS 27023A successfully reduced angiogenesis and metastasis in rat models [201].

Interestingly, integrins, transmembrane adhesion receptors responsible for the connection between cells and ECM, could provide excellent opportunities for therapeutic targeting [202]. However, the results of clinical trials with integrin inhibitors are disappointing in the existing literature. The variable integrin expression, the redundant function, the different roles depending on disease stage, and the binding of therapeutic agents by integrin-positive extracellular vesicles released from tumors are all factors preventing the impactful clinical application [203]. The role of integrins in therapy requires further investigation.

As far as the cell components of the TME are concerned, CAFs’ contribution to collagen matrix density provides a basis for their potential use as anti-tumor targets. The incorporation of anti-CAF medications in clinical protocols has shown positive effects in some cases. In endometrial cancer, high levels of Interleukin-6 are secreted by CAFs. Neutralization of Interleukin-6 reduced cell proliferation with a 2.4-fold change [30].

### 8.2. Receptor Tyrosine Kinase Inhibitors

Receptor tyrosine kinases (RTKs) are a group of transmembrane proteins functioning as essential messengers between extracellular signals and intracellular responses. Vital cellular processes like survival, proliferation, differentiation and migration are controlled by RTKs. As is readily understood, dysfunction of these proteins can dysregulate cellular processes, thereby promoting malignant behavior [204]. Importantly, several RTKs interact directly with ECM components. Integrins and ECM-bound proteoglycans can form complexes with RTKs, amplifying signaling cascades and enhancing tumor cell motility and invasion. For example, fibronectin and collagen engagement with integrins synergizes with growth factor signaling, including PDGF and VEGF pathways, to activate downstream cascades such as MAPK and PI3K/Akt [205,206]. In endometrial stromal tumors, aberrant ECM remodeling supports RTK signaling by serving as a reservoir for growth factors [205]. The intervention in the RTK pathway can be conducted through different therapeutic strategies. The function of these receptors gets suppressed by monoclonal antibodies (mAbs) and small-molecule tyrosine kinase inhibitors (TKIs) [207].

Monoclonal antibodies are valuable tools in modern oncology, providing high specificity and efficacy in the fight against a variety of tumors. Monoclonal antibodies mediate the inhibition of RTKs in two main ways: binding to the receptor’s extracellular domain and neutralizing ligands [208]. A variety of pharmaceutical agents of this kind have been recruited for endometrial cancer treatment. VEGF-R belongs to the receptor tyrosine kinase family and plays a key role in the angiogenic process, thereby contributing to tumor establishment and progression. VEGF, bound to ECM proteoglycans such as perlecan, drives angiogenesis; its inhibition by monoclonal antibodies, such as bevacizumab, reduces ECM-dependent angiogenic signaling [205,209]. It has already been tested in patients with endometrial cancer. The anti-angiogenic properties of this factor demonstrated a survival benefit, but the specific effect on stroma-derived tumors warrants further investigation [210]. Given that ECM proteins and proteoglycans act as reservoirs and co-receptors for growth factors, combining RTK inhibitors with therapies that remodel the ECM (e.g., enzymatic degradation of hyaluronan, or inhibition of collagen cross-linking by LOX inhibitors) may enhance drug penetration and reduce compensatory signaling loops. Such strategies could synergistically suppress angiogenesis and tumor–stroma interactions in endometrial stromal sarcomas.

Small-molecule tyrosine kinase inhibitors (TKIs) form a group of low molecular weight compounds able to block the function of receptor tyrosine kinase enzymes, by binding and prohibiting the receptor’s activation [211]. Platelet-Derived Growth Factors are a family of proteins binding to receptor tyrosine kinases PDGFR-α and PDGFR-β. Notably, PDGF constitutes one of the most clinically relevant RTK in endometrial stromal tumors [212,213]. Not only stimulates malignant cell survival and angiogenesis but also promotes tumor–stroma interactions through deposition of ECM proteins and fibroblast activation [205,214,215]. Expression of PDGFR-α and PDGFR-β in LG-ESS, HG-ESS, and UUS underscores the ECM–RTK interplay, since activated stromal fibroblasts deposit collagens and fibronectin that reinforce PDGF-driven oncogenic loops [216]. A variety of officially approved pharmaceutical products include PDGFR among their targets. Imatinib [217], sunitinib [218], pazopanib [219], dasatinib [220], and avapritinib [221] provide effective treatment against several different types of cancer, and their use in PDGFR-positive ESTs is promising. Further investigation of these targets and their inhibition by mAbs and small molecule TKIs is a promising field in endometrial cancer research.

### 8.3. Epigenetic Modulators

Endometrial stromal tumors commonly harbor severe epigenetic disruptions associated with dysregulated gene expression. The malignant behavior of cancer cells results from this dysfunctional cascade in many different EST cases. As previously mentioned, the most common chromosomal rearrangement among Low-Grade Endometrial Stromal Sarcoma is JAZF1–SUZ12 fusion. The PRC2 chromatin remodeling complex is significantly affected by this mutation, leading to impaired H3K27 methylation and triggering Wnt pathway hyperactivation [222]. Notably, the Wnt cascade is responsible for vital cellular processes such as cell proliferation, migration, and survival, thereby playing a leading role in cancer pathogenesis [223]. Moreover, Wnt signaling is closely regulated by the ECM: proteoglycans such as syndecans and glypicans control Wnt ligand availability and distribution [224], the SLRP, biglycan binds to LRP6, activating the Wnt pathway [57,225] while collagens and matricellular proteins influence receptor activation and downstream pathway dynamics, thereby amplifying the oncogenic effects of epigenetic dysregulation [223,224]. Consequently, correcting PRC2 dysfunction or aberrant Wnt activity has a promising therapeutic value in EST cases. PRC2 has already been suggested as a potential target in endometrial stromal sarcomas (ESSs) [226]. Moreover, another epigenetic alteration with significant clinical impact concerns histone deacetylases (HDACs). HDACs are responsible for chromatin condensation by removing acetyl groups. Dysregulation in this mechanism of gene silencing has been linked to carcinogenesis in several cancer types, including endometrial stromal sarcomas. Specifically, in ESS, a high expression pattern of HDAC1, 4, 6, 7, and 8 has been observed, and increased HDAC6 in UUS. The inhibition of these enzymes could be a promising research subject in the future, with present HDAC inhibitors like SAHA displaying a reduction in cell proliferation in ESS cultures [227]. Notably, in other models, HDAC activity not only silences tumor suppressor genes but also regulates ECM gene expression. For instance, HDAC inhibition has been linked to reduced synthesis of ECM components [228,229].

### 8.4. Immune Checkpoint Inhibitors

The immune checkpoints are the mechanisms utilized by the immune system to sustain self-tolerance and prevent hyperactivation. Two significant checkpoints involved in cancer outcomes are PD-1/PD-L1 and CTLA-4 pathways. Specifically, PD-1/PD-L1 cascade includes the Programmed Cell Death protein 1 (PD-1), a transmembrane protein expressed by activated T-cells, and PD-L1, the ligand of PD-1, which regulates immune response. The interaction between PD-1 and its ligand decreases effector T cells’ activity and increases immunosuppressive regulatory T cells, resulting in immune exhaustion [230]. The second checkpoint, cytotoxic T-lymphocyte-associated protein 4 (CTLA-4), competes with the costimulatory molecule CD28 of T-cells, inhibiting their interaction with antigen-presenting cells, leading to suppression of T-cell activity [231].

Highly immunogenic tumors commonly develop adaptive immune resistance by exploiting these checkpoints to evade immune surveillance, upregulating PD-L1 and CTLA-4 in cancer cells. These proteins constitute targets for a group of emerging pharmaceutical factors, the Immune Checkpoint Inhibitors (ICI). As expected, only immunogenic tumors are ICI responsive [232]. Stromal tumors represent a subtype of endometrial tumors with relatively low mutational burden and thus immunogenicity. However, PD-L1 and CTLA-4 are expressed in 25% and 13% of EES, respectively [172]. More aggressive cases display increased immunosensitivity, and it has been proposed that ICI has great potential in HG-ESS treatment [233].

Importantly, recent evidence suggests that the ECM also contributes to shaping immune checkpoint responses. ECM proteins such as collagens and fibronectin can form physical barriers that limit immune cell infiltration, while proteoglycans, including versican and biglycan, modulate inflammatory signaling and T-cell recruitment. Additionally, ECM remodeling enzymes, such as MMPs, regulate cytokine bioavailability [234,235,236]. These findings highlight that combining ICIs with approaches targeting ECM remodeling may improve immune cell access to tumor tissue and enhance therapeutic efficacy in endometrial stromal tumors.

### 8.5. Personalized Medicine—Genetic Profiling

Endometrial stromal tumors constitute a heterogeneous group of tumors with significant molecular complexity. Every case displays a unique genetic, molecular and histopathological profile, affecting the prognosis and therapeutic decision-making. Immunohistochemistry is one of the first steps during ESTs management. It is essential for diagnosis and distinguishing cancer subtypes based on specific markers. These biomarkers are also utilized for the estimation of disease aggressiveness and the identification of potential targets. For instance, ER and PR positive tumors are great candidates for hormonal therapy. However, more aggressive and resistant cases demand more advanced molecular assays to orchestrate more targeted approaches. Genetic profiling is required to identify fusions and other mutations, which are necessary for tumor classification and estimation of response to specialized treatments, such as epigenetic modulators and ICI [172].

Importantly, ECM-related gene signatures are increasingly recognized as informative biomarkers in cancer. Alterations in the expression of ECM components such as collagens, fibronectin, and matrix metalloproteinases, as well as proteoglycans such as versican, perlecan, and biglycan, have been correlated with disease progression, invasion, and therapeutic resistance in various malignancies [237,238], including gynecologic [239]. In ESTs, such ECM gene expression patterns may help refine patient stratification and identify those likely to benefit from RTK inhibitors or combinatorial regimens. Furthermore, a detailed understanding of the tumor’s characteristics is informative about the potential of RTK inhibitor treatment.

Modern oncology offers a variety of treatment options, transforming the protocol-based medicine into personalized healthcare, especially in a highly heterogeneous cancer type as ESTs.

## 9. Conclusions

Endometrial stromal tumors constitute a rare but clinically significant category of uterine mesenchymal neoplasms. Advances in classification have divided this group into four different subtypes: ESNs, LG-ESS, HG-ESS, and UUS. Considerable heterogeneity is observed among these types, with each one defined by distinct histopathological, immunohistochemical, and molecular profiles. This unique combination of features provides insight into the tumor’s biology, assisting with diagnosis, prognosis, and further decision-making regarding treatment options. The role of TME in disease progression is crucial. TME comprises tumor, stromal, epithelial, endothelial and immune system cells and the ECM, which contains the non-cellular components. A variety of different molecules, collagens, enzymes, proteoglycans, glycosaminoglycans, glycoproteins and other extracellular molecules have emerged as key modulators in signaling transduction, adhesion, proliferation, invasion, migration and metastasis of cancer cells, as well as angiogenesis and tumorigenesis. Given the uncertain results of current treatment options and the significant side effects they often cause, especially in high-grade cases, the need for new, efficient therapies arises. Utilizing modern knowledge of tumors’ molecular profiles, microenvironments, and ECM, some emerging treatments include targeted molecular therapies such as receptor tyrosine kinase inhibitors, epigenetic modulators, checkpoint inhibitors, and personalized medicine based on genetic profiling. This review pinpoints that further research in this promising field appears crucial and has the potential to bridge the gap between the understanding of basic tumor biology and effective, specialized treatments.

## Figures and Tables

**Table 1:** Histopathological characteristics of different endometrial stromal tumors (ESTs) types.

EST Type	Tumor’s Nature	Invasion	Cell Morphology	Mitotic Rate
ESN	Benign [4,9,10]	None	Small cells, oval nuclei, scant cytoplasm	Low
LG-ESS	Malignant [3,4,8,9]	Tong-like myometrial Possible lymphovascular	Small cells, oval nuclei, scant cytoplasm [3,9]	Low
HG-ESS	Malignant [4,8,9]	Tong-like myometrial Lymphovascular	Large, high-grade round cells Low-grade spindle cells	High
UUS	Malignant [4,8]	Destructive myometrial Lymphovascular	Epithelioid and spindled cells, multiple and unconventional nuclei	High

**Table 2:** Immunohistochemical and molecular characteristics of different endometrial stromal tumors (ESTs) types.

EST Type	ESN [4,13]	LG-ESS [4,9]	HG-ESS [4,9]	UUS [4,8]
Chromosomal rearrangement	JAZF1 SUZ12 fusion	JAZF1 SUZ12, JAZF1-PHF1, EPC1-PHF1, MEAF6-PHF1, ZC3H7-164 BCOR62, MBTD1-CXorf67	YWHAE rearrangements	Variable. JAZF1-SUZ12, YWHAE
CD10	Positive	Positive	Low or negative in round cells Occasionally positive in spindle cells	Variable
ER/PR	ERα and PR focally	ERα positive ERβ negative PR normal	Low or negative Occasionally positive in spindle cells	Variable. Positive in uniform neoplasms
WT1/β-catenin	Negative	WT1 positive β-catenin variable	WT1 low or negative	Variable
P53	Normal	Normal	Normal	Aberrant
Other molecules	SMA, vimentin, desmin positive	AR, SMA, Desmin	Cyclin D1 overexpression SMA, desmin negative	SMA, desmin variable

**Table 3:** PG expression in uterine tissues and tumors.

Proteoglycan (PG)	Normal Endometrium	Fibroids/ Leiomyomas	Hyperplasia/ Polyps	Carcinoma (EEC/ESC)
Decorin [59,67,68,69,70,71]	Present, regulates collagen fibrils	Overexpressed; longer GAG chains	Absent	Altered; loss linked to invasion/metastasis
Biglycan [37,59,60,61,70]	Present, contributes to fibrillogenesis	Highly expressed; thick collagen fibrils	Low expression	Upregulated in stroma, linked to EMT & inflammation
Lumican [59]	Present, low expression	Variable expression	Low expression	Reduced in polyps/ hyperplasia, variable in carcinoma
Fibromodulin [59]	Present	Highly expressed (proliferative phase)	Absent	Reduced/Absent
Versican [67,72,73]	Baseline expression	Overexpressed	Altered; associated with proliferation	Upregulated in tumor stroma; supports proliferation
Syndecans [67,74,75]	Baseline expression	Overexpressed	Altered expression	Overexpressed; linked to invasion and viability of cells
ECM protein-2 [67]	Baseline expression	Overexpressed	ND	Upregulated

**Table 4:** Presentation of the eight diagnostic features for uterine sarcomas as proposed by the ESGO/EURACAN/GCIG guidelines for the management of patients with uterine sarcomas. The presence of at least four features raises suspicion of sarcoma.

MRI Sequence	Features
T2-weighted imaging [132]	Heterogeneity of the solid-enhancing component Hyperintensity of the solid enhancing component
T1-weighted imaging (pre-contrast) [132]	Intra-tumoral hemorrhage
T1-weighted imaging (post-contrast) [132]	Heterogeneous enhancement Enhancing finger-like projections Ill-defined borders with the myometrium Central necrosis
Diffusion-weighted imaging (DWI) [132]	Restricted diffusion (apparent diffusion coefficient value < 0.9)

**Table 5:** Overview of the endocrine therapy used in case of low-grade endometrial stroma sarcoma.

Agent (Example)	Primary Mechanism of Action	Efficacy	Main Side Effects
Aromatase Inhibitors (AIs), Letrozole Anastrozole	Block estrogen production from peripheral tissues.	Highly effective for advanced/recurrent disease; reduces recurrence risk.	Hot flashes, joint pain, risk of bone loss.
Progestins, Megestrol Acetate (MA), Medroxyprogesterone Acetate (MPA)	Suppress endometrial growth; exert anti-estrogenic effects.	Effective as primary or adjuvant therapy; reduces recurrence.	Weight gain, fluid retention, metabolic changes.
GnRH Agonists Goserelin, Leuprolide	Inhibit ovarian function	Used mainly in premenopausal women to lower estrogen levels.	Hot flashes, risk of bone loss.
Selective Estrogen Receptor Degraders (SERDs) Fulvestrant	Bind to and degrade the Estrogen Receptor (ER).	Emerging option, particularly for tumors resistant to AIs.	Injection site pain, nausea, hot flashes.

**Table 6:** FIGO staging for uterine leiomyosarcomas and endometrial stromal sarcomas.

Stage	Definition
I	Tumor limited to uterus [140]
IA	Less than 5 cm [140]
IB	More than 5 cm [140]
II	Tumor extends beyond the uterus, within the pelvis [140]
IIA	Adnexal involvement [140]
IIB	Involvement of other pelvic tissues [140]
III	Tumor invades abdominal tissues (not just protruding into the abdomen) [140]
IIIA	One site [140]
IIIB	More than one site [140]
IIIC	Metastasis to pelvic and/or para-aortic lymph nodes [140]
IVA	Tumor invades bladder and/or rectum [140]
IVB	Distant metastasis [140]

**Table 7:** Potential targeted therapies for HG-ESS, currently under investigation.

Target/Molecular Pathway	Agent	Rationale
Angiogenesis/ VEGF	Tyrosine Kinase Inhibitors (TKI)	HG-ESS and other uterine sarcomas are often highly vascular
BCOR Gene Alterations	CDK4/6 Inhibitors MDM2 Inhibitors	HG-ESS with BCOR rearrangements frequently show co-occurring molecular changes, such as alterations in the CDK4 pathway or MDM2 amplification [173]
YWHAE::NUMT2A/B gene fusion	RAF/MEK/MA PK inhibitors Hippo/YAP- TAZ inhibitors Cyclin D1 inhibitors	Knockdown of YWHAE::NUTM2 leads to down-regulation of cell proliferation [172] YAP activation is associated with poor prognosis [174]
c-kit	c-kit inhibitors	Pazopanib and Imatinib mesylate show promising results in treating c-kit-expressing HG-ESS [172]
Immune Checkpoint	Immune Checkpoint Inhibitors (ICI)	HG-ESS is often characterized by a high degree of immune cell infiltration and can express positive predictors of immunotherapy efficacy
PI3K/AKT/mTOR	PI3K/AKT/mT OR Pathway Inhibitors	This pathway is frequently involved in cell growth and is mutated/activated in a subset of HG-ESS [175]
HER2/ERBB2	Antibody-Drug Conjugates	HER2 overexpression is reported in a small subset of mesenchymal uterine tumors [176]

## Abbreviations

The following abbreviations are used in this manuscript:

αSMA	alpha-smooth muscle actin
bFGF	basic fibroblast growth factor
CAF	carcinoma-associated fibroblasts
CCL5 or RANTES	Chemokine ligand 5
CD44	Cluster of Differentiation 44
CS	chondroitin sulfate
CSF-1	colony-stimulating factor 1
CTLA-4	cytotoxic T-lymphocyte-associated protein 4
DS	dermatan sulfate
ECM	extracellular matrix
EEC	endometrial endometrioid carcinoma
EMT	epithelial-to-mesenchymal transition
ESNs	Stromal Nodules
ESTs	Endometrial stromal tumors
FIGO	Federation of Gynecology and Obstetrics
GAGs	glycosaminoglycans
HA	Hyaluronan
HDACs	histone deacetylases
HG-ESSs	High-Grade Endometrial Stromal Sarcomas
HIPEC	hyperthermic intraperitoneal chemotherapy
HS	heparan sulfate
ICI	Immune Checkpoint Inhibitors
IL	interleukin
IL-1b	interleukin 1 beta
IL-6	interleukin 6
KS	keratan sulfate
LG-ESSs	Low-Grade Endometrial Stromal Sarcomas
LOX	lysyl oxidase
LRP6	Low-density lipoprotein receptor-related protein 6
MCP-1 or CCL2	monocyte chemotactic protein- 1
MMPs	Matrix metalloproteinases
NK	natural killer
OPN	osteopontin
PD-1	Programmed cell Death protein 1
PDGFR	Platelet-Derived Growth Factor receptor
PGs	proteoglycans
PR	progesterone receptor
ER	estrogen receptor
RHAMM	receptor for hyaluronic acid-mediated motility
RTKs	Receptor tyrosine kinases
SLRPs	small leucine-rich proteoglycans
SMA	smooth muscle actin
TADCs	tumor-associated dendritic cells
TAM	tumor-associated macrophages
TIMPs	inhibitors of metalloproteinases
TKIs	tyrosine kinase inhibitors
TME	tumor microenvironment
TNF-a	tumor necrosis factor-alpha
UCEC	uterine corpus endometrial carcinoma
UUSs	Undifferentiated Uterine Sarcomas
VEGF	vascular endothelial growth factor
